# The Glenoid Fossa’s Morphometric Investigation and Its Clinical Implications

**DOI:** 10.7759/cureus.39981

**Published:** 2023-06-05

**Authors:** Madhavi Tankala, Susmita Senapati, Shashi Shankar Behera, Satyanarayan Shamal

**Affiliations:** 1 Department of Anatomy, Kalinga Institute of Medical Sciences, Bhubaneswar, IND; 2 Department of Obstetrics and Gynaecology, Ananta Institute of Medical Sciences, Udaipur, IND

**Keywords:** glenoid cavity dimension, glenoid cavity index, glenoid notch, prosthesis, shoulder arthroplasty

## Abstract

Background

The inconsistent morphology of the scapula is based on variable dimensions of its glenoid cavity, in addition to its broadened and truncated lateral angle. Its variable shapes are attributed to the spinoglenoid cavity (superior and posterior aspect of the scapula), which appears oval, inverted comma-shaped, and piriform (pear-shaped). Traumatic conditions often lead to glenoid dislocation/fracture. The precise administration of total shoulder arthroplasty with glenoid component adjustment warrants a comprehensive knowledge of scapular morphology. This study aims to assess the glenoid cavity/scapula shapes (anthropometric assessment) among individuals located in Odisha, India.

Methods

This cross-sectional analysis was undertaken on 74 left-sided and 70 right-sided, dry, and undeteriorated scapulae of human adult specimens obtained from the anatomy department irrespective of their gender and age.

Results

The glenoid cavity was most commonly inverted comma-shaped (34.02%) and pear-shaped (48.61%) while 17.36% of scapulae had oval-shaped glenoid cavities. The mean scapular breadth and length dimensions were 98.12±7.87mm and 135.76±12.85mm, respectively. Statistically insignificant bilateral variations were observed between the glenoid cavity index (mean value: 68.44±7.98%), glenoid diameter-2 (anteroposterior; mean value: 16.17±2.24mm), glenoid diameter-1 (anteroposterior; mean value: 22.67±1.53mm), and glenoid diameter (superoinferior; mean value: 36.03±2.15mm).

Conclusion

The size and shape of the glenoid cavity are directly associated with the dislocation of the shoulder joint and may disturb the results of total shoulder arthroplasty and rotator cuff surgeries. The current study analyzed the morphological types and diameters of the glenoid cavity in the scapulae to improve efficiency and lessen the failure proportions in shoulder arthroplasty. The study shows that morphological measurement of scapulae plays a vital role in the effective maintenance of posture and shoulder functions.

## Introduction

Globally, there is a wide range of variations in the scapula based on ontogenic, phylogenic, and racial differences, and it’s one of the important bones in anatomical research [1]. Further, analyzing the dimensions of the scapula is useful in studying the pathological mechanism of various upper musculoskeletal injuries [2].

The glenoid cavity or fossa has a pyriform articular surface constituting the shallow lateral angle of the scapula. This anatomical region of the upper extremity is predisposed to frequent injuries due to its wider and longer vertical diameter. The dislocation/fracture of the glenoid cavity is the most frequently reported condition following shoulder trauma; total shoulder replacement is the method of choice to rearrange the anterior muscles, perform the overlapping repair, and undertake capsular reinforcement during the labrum reconstruction [3].

Various shapes of the glenoid cavity have been described based on the presence of a notch on the anterior glenoid rim. It has been found that if the notch is distinct, then the glenoid labrum is not fixed to the bony margin of the notch but bridges the notch itself. This could make the shoulder joint less resistant to dislocating forces [4]. However, substantial variations in the glenoid cavity’s morphology reciprocate with the glenoid notch’s absence/presence on the rim of the anterior glenoid. These anatomical variations are responsible for marked differences in the glenoid cavity’s shapes between individuals. Accordingly, the glenoid cavity may acquire the shape of an inverted comma, ovoid, pear, or teardrop. The glenoid notch appears more prominent during the initial developmental stages of the upper extremity bones; with a separate ossification center over it, the glenoid notch marks the articular surface of the shoulder, including the junction of the scapula and the coracoid [5]. The rotator cuff tears with full thickness indicate the glenoid inclination [6]. Accordingly, evidence reveals the prognostic significance of the glenoid cavity’s dimensions in terms of determining primary glenohumeral osteoarthritis, rotator cuff disease, and shoulder dislocation; in addition, shoulder arthroplasty is predominantly guided by the dimensions of the glenoid components [7]. A glenoid osteochondral defect occurs often as a result of acute trauma, leading to intraarticular bodies, labral tears, and instability [8]. Studies reveal a 20% incidence of the Bankart lesion in unilateral shoulder instability cases, reported via roentgenograms [8]. The shape, inclination, width, and height of the glenoid fossa help determine the placement and design of the corresponding prosthesis. Importantly, the intraoperative implantation procedures, instrumentation, and design of the prosthesis vary considerably due to marked differences in these attributes among the individuals. This study aimed to perform the glenoid cavity’s morphometric assessment to understand its implications in total shoulder arthroplasty in Odisha, India. 

## Materials and methods

This cross-sectional study was conducted from April to December, 2022, and investigated the anatomical parameters of 144 (left-sided = 74, right-sided = 70) undeteriorated dry scapulae of humans obtained from the Department of Anatomy, Kalinga Institute of Medical Sciences, Odisha, India. The study was approved by the Institutional Ethical Clearance Committee of Kalinga Institute of Medical Sciences (approval number: KIIT/KIMS/552/2021). This study did not include scapulae with structural deterioration and investigated only those with intact and prominent anatomical attributes. The assessment of anatomical characteristics relied on a 0.01 mm digital vernier-type calliper; entire assessments of the anatomical parameters were undertaken in millimeters. 

The glenoid cavity's shape

A lead pencil and white paper were used to effectively trace the glenoid cavity's dimensions; the tracings revealed the oval, inverted comma type, and pear shape of the glenoid cavity.

Metric Parameters

**Figure 1:**
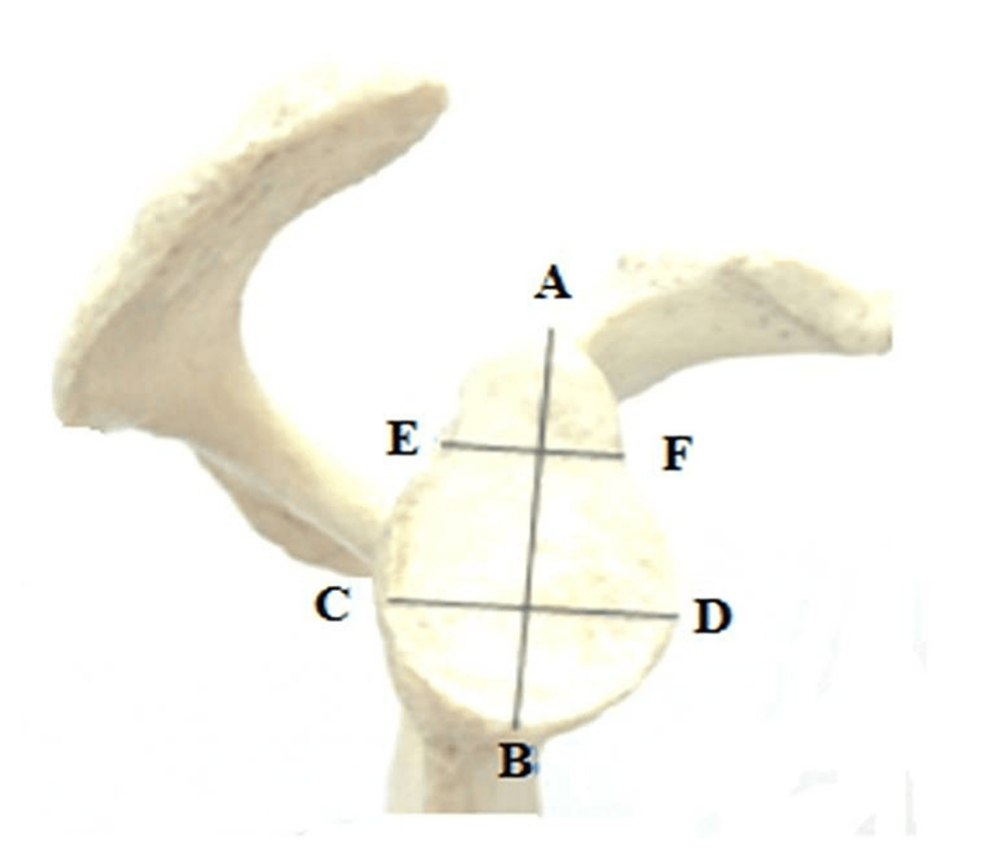
Measurements of glenoid cavity Image source: Singh et al., 2019 [9]

The maximum length of the scapula was calculated by measuring the distance between the inferior (point B) and superior (point A) angles’ summits. The maximum breadth of the scapula was assessed by tracing the distance between points D and C on the junctions of the glenoid cavity's posterior (middle region) and medial borders with the spine. The superoinferior diameter was evaluated by measuring the distance between points F and E, indicating the glenoid margin’s inferior-most region and the supraglenoid tubercle’s prominent parts (Figure 1). 

Diameter 1 of the glenoid (anteroposterior part (AP)-1) has been taken as the maximum breadth of the articular margin of the glenoid cavity at 90^o^ to the glenoid cavity height. Diameter 2 of the glenoid (anteroposterior part (AP)-2) has been taken as the anteroposterior diameter of the upper half of the glenoid cavity at its mid-point [9].

Glenoid cavity index (GCI)

The following formula guided the GCI assessment.

GCI=Glenoid diameter 1 (anteroposterior part) ÷ glenoid diameter (superoinferior part) × 100

Data assessment

The data analysis was undertaken by IBM SPSS Statistics for Windows, Version 24.0 (Released 2016; IBM Corp., Armonk, New York, United States). While percentages were retrieved from the categorical variables, the standard deviation and mean values expressed the continuous variables. The left and right portions of the glenoid cavity were analyzed by the paired t-test. The statistical significance of outcomes was indicated by the corresponding p-value reference (<0.05).

## Results

The findings of this study revealed that pear shape was observed to be 23.8% on the right side and 26.64% on the left side, inverted coma shape was observed to be 17.5% on the right side and 17.76 on the left side, and oval shape was found to be 7.7% on the right side and 10.6 on the left side of the glenoid fossa, respectively (Table 1). The distribution of shapes of glenoid fossa was found to be highest with 48.61% for the pear shape, followed by 34.02% for the inverted coma shape, and finally 17.36% for the oval shape in the 144 samples.

**Table 1:** The patterns of the glenoid fossa shapes/structures

Structure	Right	Left	Total
Pear	34 (23.8%)	36 (26.64%)	70 (48.61%)
Inverted comma	25 (17.5%)	24 (17.76%)	49 (34.02%)
Oval	11 (7.7%)	14 (10.36%)	25 (17.36%)
Total	70 (100%)	74 (100%)	144 (100%)

The mean length of the scapula is observed as 135.76±12.85 mm on the right side and 134.11±12.92 mm on the left side. Also, the mean breadth of the scapula is observed as 98.12±7.87 mm on the right side and 97.28±8.12 mm on the left side. There was no statistical difference observed for the dimensions of the scapular. The p-value was found to be statistically insignificant (p>0.05) (Table 2).

**Table 2:** The dimensions of the bilateral scapula

Dimensions (in mm)	Right scapula	Left scapula	P-value
Scapula length	135.76±12.85	134.12±11.92	0.12
Scapula breadth	98.12±7.87	97.28±8.12	0.76

The means of the glenoid diameter 2 mm (anteroposterior part), glenoid diameter 1 mm (anteroposterior part), and glenoid diameter (superoinferior part) were 17.30±1.16 mm, 22.67±1.53 mm, and 36.03±2.15 mm, respectively. No statistically significant differences were observed in the scapula dimensions bilaterally (p>0.05) (Table 3). 

**Table 3:** The glenoid cavity characteristics

Parameters	Right Side	Left Side	P-value
Superoinferior glenoid diameter (mm)	36.03±2.15	35.52±2.12	0.43
Anteroposterior glenoid diameter 1 (mm)	22.67±1.53	22.59±1.47	0.64
Anteroposterior glenoid diameter 2 (mm)	17.30±1.16	17±1.34	0.42

The mean GCI were 68.44±7.98% and 68.84±7.64 across the right and left locations, respectively, with statistically insignificant bilateral variations (p=0.26) (Table 4). 

**Table 4:** The glenoid cavity index

Parameter	Right Side	Left Side	P-value
Mean glenoid cavity index	68.44±7.98	68.84±7.64	0.26

## Discussion

A range of studies across the globe provides mounting evidence on the scapular morphometry of individuals from different races and geographical locations. The morphometry outcomes concerning the glenoid cavity/scapula are derived from live radiographic assessments, the dry scapula's structural analysis, and embalmed cadavers’ straightforward measurements. The findings from this study were obtained from the morphometric assessment of the dry scapulae of the human cadavers. Substantial variations and similarities in the glenoid cavity/scapula dimensions were recorded in this study and were supported by the previous evidence. The inverted comma-shaped and pear-shaped glenoid fossae were observed in 34.02% and 48.61% of cases, respectively. Findings from this study concorded with the outcomes of Kalra et al. and Rajput et al. that indicated the highest occurrences of inverted comma and pear structures of the glenoid cavity [7,10]. These findings revealed the greatest incidence of pear structures followed by the inverted comma shapes of the glenoid fossa. Contrarily, the findings of Amin and Hassan indicated the highest incidence of inverted comma shapes followed by pear structures [11].

Previously, Prescher and Coskun et al. also conducted research on these parameters and categorized the shapes of the glenoid cavity into pear, inverted coma, and oval. These dimensions will provide significant information regarding the designing and fitting of glenoid dimensions for shoulder arthroplasty [12,13].

Outcomes from this study revealed a high similarity with findings from Patel et al. regarding the scapula dimensions (i.e., mean length) (135.76±12.85 mm vs. 119.63±8.81 mm (females)/136.03±11.49 mm (males)) [14]. Slight differences were reported between our study outcomes and the findings of Azhagiri et al. (149.58 mm) about regions in South India [15]. Alternatively, higher scapular dimensions were revealed in a study conducted in Egypt (151.16±10.32 mm) [11]. Marked variations across the populations are possibly responsible for the reported differences in scapular dimensions.

Findings from this study concerning the scapular breadth (mean value: 98.12±7.87 mm) concorded with the outcomes of Singal et al. (mean value: 96.4±7 mm) [16]. Outcomes of El-Din et al. defied these results by affirming a comparatively higher scapular breadth (mean value: 107.22±9.74 mm) [11].

Statistically insignificant differences were observed between the superior-inferior diameters of the glenoid fossa on the left and right sides, respectively (35.52±2.12 mm vs. 36.03±2.15 mm; p>0.05). These findings matched the outcomes of Ankush Rao and Dombe (36.52±4.12 mm vs. 37.03±3.55 mm; p>0.05) [17] and Raaj et al. (31.6 mm vs. 33.1 mm; p>0.05) [18]. Importantly, evidence from both studies indicates greater right superior-inferior glenoid diameter in comparison to the contralateral side. Contrarily, the outcomes of El-Din et al. reveal greater bilateral superior-inferior glenoid diameters compared to the present study; however, the left diameter superseded the right location (39.01±2.49 mm vs. 38.88±2.63 mm) [11].

Statistically insignificant differences were recorded between the left and right-sided glenoid diameters 1 (22.59±1.47 mm vs. 22.67±1.53 mm) and 2 (17±1.34 mm vs. 17.30±1 mm, both anteroposterior aspects, respectively). 

The left and right-sided GCIs (mean values) were 68.84±7.64 mm and 68.44±7.98 mm, respectively, and their differences lacked statistical significance (p>0.05). Findings of Polguj et al. and Dhindsa et al. revealed the combined (72.35±5.55 mm) and mean (68.59±4.36 mm (left); 70.37±4.08 mm (right)) GCIs, respectively [1,19]. However, the findings of Hassanein revealed lower dimensions of the left compared to the right side GCI (mean values: 76.71±8.37 vs. 73.67±9.08) [20]. These findings provided evidence concerning the higher dimensions of the glenoid fossa in comparison to our outcomes. 

Limitations

The major limitation of the study is the low sample size. We also did not do the morphometric analysis based on gender-based differences. In addition, we did not aim for three-dimensional morphometry analysis, which would have emerged as an additional value.

## Conclusions

The study shows that the right and left bone parameters must be evaluated. Further, morphological measurement of scapulae orchestrates a vital role in the effective maintenance of posture and shoulder functions. Outcomes from this study will improve the decision-making regarding shoulder arthroplasty, in terms of appropriate prosthesis selections and placement. Dissimilarities in the size and shape of the glenoid cavity that were detected in the present study will help orthopaedic surgeons to know the shoulder pathology better and to select the appropriate size of the glenoid module for shoulder arthroplasty and related shoulder ailments.
